# Acupuncture for dry eye: a multicentre randomised controlled trial with active comparison intervention (artificial tear drop) using a mixed method approach protocol

**DOI:** 10.1186/1745-6215-11-107

**Published:** 2010-11-16

**Authors:** Tae-Hun Kim, Jung Won Kang, Kun Hyung Kim, Kyung-Won Kang, Mi-Suk Shin, So-Young Jung, Ae-Ran Kim, Hee-Jung Jung, Seung-Deok Lee, Jin-Bong Choi, Sun-Mi Choi

**Affiliations:** 1 Acupuncture, Moxibustion and Meridian Research Centre, Korea Institute of Oriental Medicine, Daejeon, South Korea; 2 Department of Acupuncture and Moxibustion, Dongguk University, Goyang, South Korea; 3 Department of Oriental Rehabilitation Medicine, Dongshin University, Gwangju, South Korea

## Abstract

**Background:**

Previous studies of acupuncture show favourable results for both subjective and objective outcomes of dry eye. However, firm conclusions could not be drawn from these studies because the quality of the trials was too low to establish concrete evidence. Therefore, this study was designed both to avoid the flaws of the existing trials and to assess the effectiveness, cost-effectiveness and qualitative characteristics of acupuncture treatment for dry eye.

**Methods/design:**

One hundred fifty participants with dry eye will be recruited into three independent hospitals from different areas: Korea Institute of Oriental Medicine, DongGuk University Ilsan Oriental Hospital and Dongshin University Gwangju Oriental Hospital. The number of participants required was calculated from the data of a previous, relevant study. These patients will be randomly allocated into acupuncture treatment or artificial tear groups. Either 17 acupuncture points (bilateral BL2, GB14, TE 23, Ex1, ST1, GB20, LI4, LI11 and single GV23) will be used 3 times a week or disposable artificial tear drops (Refresh Plus^®^, ALLERGAN) will be provided for use at least once a day for 4 weeks. The ocular surface disease index (OSDI), tear film break-up time (TFBUT), Schirmer I test, visual analogue scale (VAS) for self-assessment of ocular discomfort, general assessment (by both acupuncture practitioners and participants) and quality of life (QOL) through the Measure Yourself Medical Outcome Profile-2 (MYMOP-2) will be assessed for approximately 3-months for each study participant. In addition, qualitative study and cost-effectiveness of acupuncture treatment will be conducted.

**Trial registration:**

ClinicalTrials.gov (Identifier: NCT01105221).

## Background

Dry eye is not a life-threatening disease, but it is one of the most common and irritating conditions in ophthalmology. Because of recent data on both the pathological mechanism and aetiology, the definition of dry eye has been changed from simple ocular discomfort (related to the deficiency or hyper-evaporation of tears) to a multifactorial disease (with ocular discomfort and visual disturbances, which are related to tear-film instability and both the hyper-osmolarity and chronic inflammation of the ocular surface) [1]. The overall prevalence has been reported to be between 5 and 35 percent in different populations [2]. The incidence is now increasing more rapidly than other ophthalmologic diseases such as cataracts, retinal disease and glaucoma [3]. This may be related to an increase in average life expectancies, environmental pollution, and adverse events of various ophthalmologic interventions including LASIK surgeries and medications (including antihistamines, which serve as a major risk factor for dry eye) [2].

Acupuncture is a widely used treatment modality for various conditions, including ophthalmologic diseases [4]. It is generally accepted as an effective treatment option in clinical practices. However, neither its efficacy nor its safety is well established. This is primarily because the quality of the clinical trials that assess acupuncture is too low to draw definitive conclusions. Well-designed, rigorous research is needed to establish the evidence for wider usage of acupuncture, including for the treatment of dry eye [5].

In acupuncture research, selecting an appropriate control (including sham acupuncture) is a significant challenge to overcome. Several types of sham acupuncture methods have been developed, but it is not easy to maintain effective masking for participants when using these modalities. It is also difficult to exert a placebo effect that is distinct from real (verum) acupuncture [6]. In addition, a recent review about acupuncture research reported that sham acupuncture has a tendency to show equivalent efficacy compared to verum acupuncture [7]. Therefore, it may be easy to draw a hasty conclusion (i.e. that acupuncture has either only a small or no specific effect) in sham-controlled clinical trial designs. Comparative-effectiveness studies using either usual care or standard treatment groups has its own value because it reflects real-world clinical conditions rather than research conducted under more ideal experimental conditions [8,9].

When we consider acupuncture as an effective intervention for dry eye, it becomes important to question both what components would be relevant for evaluating therapeutic effects and what benefits would be gained from treatment [10]. Recent research on complementary and alternative medicine examines effectiveness by using mixed-method approaches to show both the qualitative factors and the economic values of the interventions [8,11].

With this in mind, we designed a multicentre, randomised, controlled trial that compares acupuncture treatment with an artificial tear drop treatment (as an active control). We also added mixed-method analyses for both the cost-effectiveness and the qualitative study for a mixed method approach.

### Study aims

The primary aim of this study is to determine the effectiveness of acupuncture treatment for dry eye. The null hypothesis is that changes in the ocular surface disease index (OSDI) are equal in both the acupuncture treatment group and the artificial tear drop group after 4 weeks of treatment.

## Methods/Design

**Figure 1:**
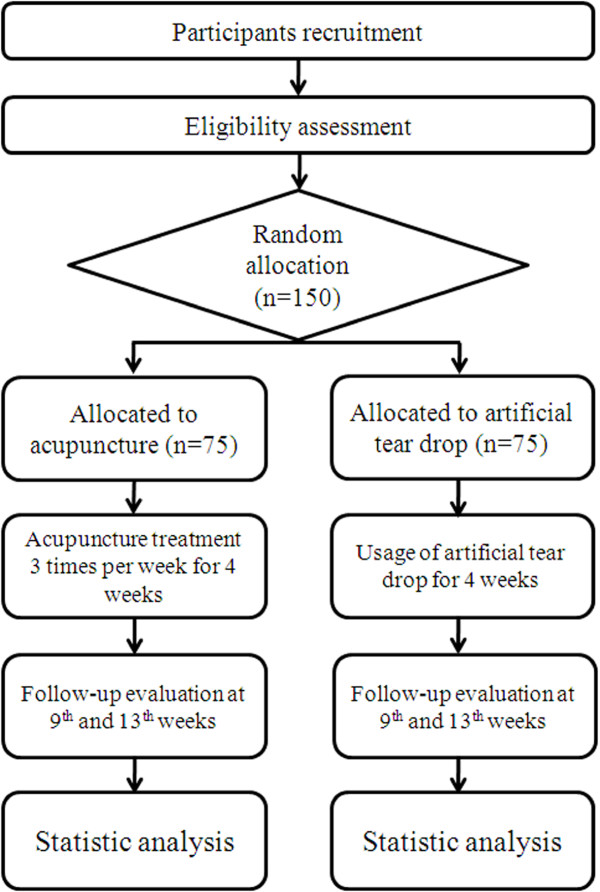
**Study Flow Chart**.

This study is a randomised, active-intervention controlled (using artificial tear drops), multicentre trial. Three clinical research centres in Korea will participate in this trial: Korea Institute of Oriental Medicine (Daejeon University Hospital), DongGuk University Ilsan Oriental Hospital and Dongshin University Gwangju Oriental Hospital. Eligible participants will be randomly allocated into either of the two groups (acupuncture or artificial tear drop) and will receive treatment for 4 weeks. Ophthalmologic tests and questionnaires for both ocular symptoms and quality of life will be assessed for up to 13 weeks after the first visit (Figure 1). Additional studies will proceed with the main outcome analyses (cost-effectiveness and qualitative factors).

### Eligibility Criteria

We will include patients who have dry eye symptoms (e.g. ocular itching, foreign body sensation, burning, pain and dryness, visual disturbance, ocular redness and sensation of tearing in a single eye or in both eyes) of at least moderate severity (i.e. tear film break-up time [TFBUT] is below 10 seconds and Schirmer I test result is below 10 mm/5 minutes) [12]. Participants will be excluded if they have prominent pathological changes of the eyelid, eyelashes, eyeball or eye accessories due to acute infection; Stevens-Johnson syndrome; Vitamin A deficiency; external injuries; a history of surgical operation related to the eye in the previous three months; punctual occlusion; sequelae of facial palsy; usage of anti-inflammatory eye drops within two weeks before entry; pregnancy; or usage of herbal medicine, acupuncture, or cupping therapy within 1 month before entry. In addition, usage of contact lenses within 2 weeks before entry, and throughout the study, is prohibited. The eligibility were assessed by physicians and ophthalmologists [13].

### Sample size

The results from a previous study were used to calculate the sample size [13]. The mean difference and standard deviation (SD) of OSDI after acupuncture treatment were 17.61 and 15.61, respectively. According to another clinical trial with artificial tear drop, the supposed mean and SD of the artificial tear group were 11.3 and 6.3, respectively [14]. A two-sided 5% significance level and 80% power were considered, and the following equation was used:

$$n=\frac{2\times {\left(z_{\alpha /2}+z_{\beta }\right)}^{2}\times \sigma^{2}}{\delta^{2}}$$

*z*_*α*/2 _= *z*_0.025 _= 1.96; *z*_1-*β *_= *z*_0.8 _= 0.84

Approximately 60 participants in each group were calculated to be required. Therefore, estimating a 20% dropout rate, 75 participants will be recruited into each group. Among the 3 participating centres, all located in Korea, approximately 50 participants will be assigned to each to ensure an even distribution.

### Randomisation and Allocation Concealment

Random numbers will be generated by a computerised random-number generator through the block-randomisation method of the SAS package (Version 9.1.3; SAS institute Inc.) for sequence generation. A clinical research associate (CRA) will make opaque blinded assignment envelopes (with consecutive numbers) and deliver them to each participating centre. The block size and treatment-assignment table will not be available to the researchers until the end of the study.

Random allocation will be performed at the second visit for each participant. According to the number assigned to a participant, the corresponding envelope will be opened in front of the participant to reveal treatment. Either acupuncture or artificial tear drop treatments will be initiated at the second visit after the assignment. Objective outcomes (i.e. Schimer test and TFBUT) will be examined by different ophthalmologists, while self-reported outcomes (e.g. OSDI, visual analogue scale (VAS), quality of life (QOL), and general assessment) will be assessed by different outcome assessors.

### Intervention

#### Acupuncture treatment

Certified acupuncture practitioners who have a minimum of 7 years of training and 3 years of clinical experience will perform the acupuncture treatments. They will take a one-day training course for this trial. This course will cover the study protocol, techniques for acupuncture treatment, the recording method for the clinical record form (CRF), and basic information about clinical research, a researcher's duty and research monitoring. The relationship between patients and practitioners can be an important factor when assessing the outcomes of acupuncture research [15]. Therefore, interactions between acupuncture practitioners and participants will be strictly limited, except when reporting adverse events.

Based on a literature review and a textbook about acupuncture for ophthalmologic diseases or dry eye [16], acupuncture points were chosen by an expert committee comprised of professors and researchers that specialise in acupuncture and ophthalmology of traditional Korean medicine. Seventeen acupuncture points (bilateral BL2, GB14, TE 23, Ex1, ST1, GB20, LI4, and LI11 and single GV23) will be located according to the WHO Standard Acupuncture Point Locations in the Western Pacific Region [17]. These will then be treated with 0.20*30 mm disposable acupuncture needles (Dongbang Co., Korea). The '*deqi*' sensation will be induced by twisting acupuncture and needles will be retained for 20 minutes before removal. Participants will have acupuncture treatments three times per week for four weeks.

#### Active comparator group using artificial tear drops

Artificial tear is one of the first choices in treatment for dry eye. Because the strategy for treating dry eye is to ease ocular discomfort and to improve quality of life, and to recover the ocular surface from chronic inflammation and tear film instability [18], artificial tear can be recommended as an active treatment. According to a survey report of ophthalmologists, preservative-free artificial tear was the most frequently selected medication for moderate to severe dry eye patients among various treatment modalities in Korea [19]. In this context, we chose preservative-free artificial tear as an active comparator intervention in this trial.

Participants allocated to this group will be offered preservative-free and one-day-use artificial tear drops (Refresh Plus^®^, ALLERGAN) and then educated on how to use them. According to the participants' discomfort from dry eye symptoms, it is recommended that the tear drops can be used at least once a day for four weeks. A diary of both the frequency and quantity of drops used will be collected at every visit.

In both groups, other treatments for dry eye will be forbidden during the study period. These other treatments include different types of artificial tear drops, drugs, supplements (e.g. cyclosporine, corticosteroids, biological tear substitute, and oestrogen), and alternative treatments (e.g. herbal medicine, acupuncture, and cupping therapy). However, participants will be allowed to use any kind of treatment for dry eye during the follow-up period. The usage of these treatments will be reported during the follow-up period.

### Primary outcome measurement

The OSDI is a questionnaire that consists of twelve questions about ocular irritation and the effect of dry eye on vision [20]. It will be assessed at the first, third, fifth, ninth and thirteenth week. For every question, participants will provide a score between zero and four, where zero equals "none of the time" and four equals "all of the time". OSDI scores will be calculated according to the following formula: OSDI = [(sum of scores for all questions answered)*100]/[(total number of questions answered)*4]. The Korean version of OSDI has not been validated yet. However, there is no available tool for evaluating dry eye that validation test for translation was finished about in Korea. So we will use the Korean version of OSDI which has been widely used for practice and research since 2006, in spite of these limitations [13,21,22].

### Secondary outcome measurement

A VAS for self assessment of ocular discomfort will be reported by participants. Ocular symptoms related to dry eye (e.g. ocular itching, foreign body sensation, burning, pain and dryness, blurred vision, sensation of photophobia, ocular redness, and sensations of tearing) will all be reported using a standard 100-mm VAS scale.

QOL questionnaire of the Measure Yourself Medical Outcome Profile-2 (MYMOP-2) will be used to assess dry-eye-related QOL [23,24]. A seven-point Likert scale (from zero-'Excellent' to six-'Worst') will be used. The questionnaire is: "During last week, how would you express your quality of life related to dry eye overall?" The VAS and questionnaire for QOL will be assessed at the first, third, fifth, ninth and thirteenth week.

The Schirmer I test (with anaesthesia) is a diagnostic method to measure the basic quantity of tear secretion [25]. After application of local anaesthesia, Schirmer test paper (Color BarTM Eagle Vision, USA) will be placed in the lateral third of the lower eyelid for five minutes while the participants' eyes are closed.

Tear film break-up time (TFBUT) is a method for observing tear film stability [26,27]. Sodium fluorescein (2.5%) will be applied to both eyes, and the tear break-up time (i.e. the interval between the last complete blink and the first appearance of a dry spot or disruption in the tear film) will be measured. Both the Schimer test and TFBUT will be assessed at the first, fifth and thirteenth week.

Acupuncture practitioners and participants will evaluate general improvement of dry eye-related symptoms using a five-grade scale: excellent, good, fair, poor and aggravation. This will be assessed after the end of the treatment period (the fifth week).

### Additional study

A nested, questionnaire-based, qualitative study will be conducted to explore the participants' reason for enrolling in this randomized controlled trial, experiences of dry-eye, and perceived changes after either acupuncture or artificial tear drop treatment. A questionnaire with open-ended questions will be distributed to all participants at the first, fifth and ninth week. They will be encouraged to write their responses in a comfortable environment. At baseline, patients in both groups will be asked how dye eye affects their lives and why they participated in this trial. At the fifth week, they will be asked how they feel after acupuncture treatment (or artificial tear drop treatment) and how they feel about being provided acupuncture treatment (or artificial tear drop treatment). At the ninth week, they will be asked how dry eye currently affects their lives and whether there was any change in their daily lives after treatment (acupuncture or artificial tear drops). Completed questionnaires will be collected at the next visit. Data will be analysed by a systematic text condensation method using the patients' written responses.

Cost-effectiveness will be analysed using the OSDI scores and comparing the cost of acupuncture with artificial tear drops for the two groups during the four-week treatment period. Additionally, the cost of dry-eye and other treatments (including acupuncture) for all of the participants during the follow-up period will be recorded and analysed.

### Statistical analysis

Statistical analysis will be conducted on an intention-to-treat basis with a 95% confidence interval using the SAS statistical package (SAS^® ^Version 9.1, SAS institute. Inc., Cary, NC). Missing values of drop-out participants will be imputed by the last observation carried forward (LOCF) method. Baseline characteristics will be displayed as the mean ± SD for continuous data or n (%) for categorical data. Because patient expectations may affect the treatment outcome [28], patient expectations (reported on a nine-point Likert scale before assignment of intervention) will also be compared between the two groups.

For analysis of baseline characteristics, either two-sample t tests or Wilcoxon rank sum tests for continuous data and Chi-squared tests or Fisher's exact tests for categorical data will be conducted after the test for normality. ANCOVA (Analysis of Covariance) will be used to test whether outcome measures (OSDI score, TFBUT, Schimer test result, QOL, VAS for self-assessment of ocular discomfort) differ between conditions when controlling for baseline and other covariates (including sites). Also, repeated measure analysis of variance will be performed to show changes in trends.

### Adverse events

All unexpected responses related to acupuncture treatment or use of artificial tear drops will either be reported to the investigators by participants or be examined by acupuncture practitioners. Local, general and psychological adverse events are possible as a result of acupuncture treatments [13]. Irritability, pain and redness of eyes, headache, and blurred vision are recognised as common adverse reactions to artificial tear drops. If serious adverse events (SAE) occur, experimental treatments will be stopped immediately, and appropriate treatments will be offered. The type and frequency of adverse events will be reported for each group.

### Ethics

This research protocol was approved by the institutional review board (IRB) of each trial centre (Daejeon University Hospital, Dongguk University Ilsan Oriental Hospital and Dongshin University Gwangju Oriental Hospital). Every participant will give informed consent before the start of this trial.

## List of abbreviations

ANCOVA: analysis of covariance; CRA: clinical research associate; CRF: clinical record form; IRB: the institutional review board; LOCF: last observation carried forward; MYMOP-2: Measure Yourself Medical Outcome Profile-2; OSDI: ocular surface disease index; TFBUT: tear film break-up time; VAS: visual analogue scale; QOL: quality of life

## Competing interests

The authors declare that they have no competing interests.

## Authors' contributions

THK wrote the study protocol and drafted this manuscript. JWK participated in study design and critical revision of the manuscript. KHK participated in the design of qualitative study assessments and in critical revision of the manuscript. KWK participated in the design of the statistical analysis and the cost-effectiveness analysis. MSS participated in the analysis of ophthalmologic test results. Both SYJ and ARK participated in assessing outcomes in the outcome assessment. JHJ conducted the sequence generation and monitored the participating clinical research centres. Both SDL and JBC helped draft the manuscript. SMC was the general supervisor for this research and participated in both the study design and critical revision of the manuscript. All authors read and approved the final manuscript.
